# Chronic interleukin-6 mediated neuroinflammation decreases anxiety, and impaires spatial memory in aged female mice

**DOI:** 10.3389/fnins.2023.1267818

**Published:** 2023-11-23

**Authors:** Ingrid Marguerite Wagnon, Lillian Jocelyn Jabur, Garry Niedermayer, Gerald Münch, Tim Karl, Rose Chesworth, Erika Gyengesi

**Affiliations:** ^1^Department of Neuropharmacology, School of Medicine, Western Sydney University, Campbelltown, NSW, Australia; ^2^School of Science, Western Sydney University, Campbelltown, NSW, Australia; ^3^Department of Behavioral Neuroscience, School of Medicine, Western Sydney University, Campbelltown, NSW, Australia

**Keywords:** chronic neuroinflammation, behavioral phenotyping, Barnes maze, RT-qPCR, mRNA, learning and memory, anxiety, interleukin-6

## Abstract

**Introduction:**

Neuroinflammation is a common feature of many psychiatric disorders as well as a common underlying mechanism of neurodegenerative diseases. Sex has been shown to strongly influence the development as well as the clinical expression of these pathologies. However, there is still a neglect regarding the consideration of sex effects in rodent experiments, and a substantial underrepresentation of females in studies. This work set out to expand our knowledge of neuroinflammatory mechanisms in female mice, at both a behavioral and molecular level.

**Methods:**

This study used GFAP-IL6 mice, a model of chronic neuroinflammation, in which interleukin-6 (IL6) is overexpressed in the central nervous system under the control of the glial fibrillary acidic protein (GFAP) promoter. We evaluated aged (11-15-month-old) wild type-like (WT) and GFAP-IL6 female mice in behavioral tests assessing anxiety (elevated plus-maze, EPM, Light/dark box), and spatial learning and memory (Y-maze, YM and Barnes Maze, BM) and associative learning (fear conditioning, FC). We also examined gene expression of markers linked to neuroinflammation, neurodegeneration and neurotransmission via RT-qPCR in brain regions involved in motor control, anxiety, learning and memory.

**Results:**

Female GFAP-IL6 mice exhibited reduced anxiety-like behavior in the EPM, and hypolocomotion in the light-dark test and EPM. Short-term memory impairment was evident in the YM but associative learning in FC was intact in GFAP-IL6 mice, suggesting domain-specific cognitive deficits in female GFAP-IL6 mice. In the BM, all mice showed intact learning and memory, but GFAP-IL6 mice exhibited higher latencies to enter the escape hole than WT mice. We analyzed the search strategy and found differences in the way GFAP-IL6 mice searched for the escape hole compared to WTs. RT-qPCR showed increased mRNA levels for molecules involved in pro-inflammatory pathways in the cerebellum, motor cortex, hippocampus, and amygdala in GFAP-IL6 mice. Of the regions examined, the cerebellum and the hippocampus showed upregulation of neuroinflammatory makers as well as dysregulation of glutamatergic and GABAergic neurotransmission gene expression in GFAP-IL6 mice compared to WTs.

**Conclusion:**

In conclusion, we showed that chronic neuroinflammation via IL6 overexpression in aged female mice led to a less anxious-like phenotype, hypolocomotion and impaired intermediate-term spatial learning and memory in the YM.

## 1 Introduction

Neuroinflammation is a common feature of many neurodegenerative diseases such as Alzheimer’s disease (AD), Parkinson’s disease (PD) or amyotrophic lateral sclerosis (ALS) (Kempuraj et al., 2016; Liu and Wang, 2017) but also appears to be involved in numerous psychiatric disorders such as depression (Barbosa et al., 2012; Brites and Fernandes, 2015; Troubat et al., 2020), bipolar disorder (Stertz et al., 2013; Haarman et al., 2014), schizophrenia (Doorduin et al., 2009; Monji et al., 2013; Aricioglu et al., 2016) and autism spectrum disorder (ASD) (Matta et al., 2019; Liao et al., 2020). A common feature across these disorders is the impairment of cognitive function, suggesting there may be a link between neuroinflammation and altered cognition.

A sexual dimorphism is observed for most of neurodegenerative, neurodevelopmental, or neuropsychiatric disorders (Abel et al., 2010; Pinares-Garcia et al., 2018; Ullah et al., 2019; Lopez-Lee et al., 2022). While it has been clearly established that sex has an influence on the prevalence, the progression, or even the behavioral outcomes of these disorders, the reasons are not completely yet understood. As neuroinflammation is a common underlying mechanism, maybe part of the answer can be found by looking at the main actors of neuroinflammation: microglia and astrocytes. Evidence of sexual dimorphism of glial cells has been shown and gathered mostly in rodents. Depending on the sex, there is a variation of the number, the morphology, the immune molecule load, and the transcriptomic profile of the microglia that also depends on the brain region and the age of the animal (Hanamsagar and Bilbo, 2016; Villa et al., 2018; Kodama and Gan, 2019; Lopez-Lee et al., 2022). Astrocytes also present with sexual dimorphism (Acaz-Fonseca et al., 2016) as shown in rodent models with differences in number and morphology of astrocytes in some brain regions (Johnson et al., 2008; Crespo-Castrillo and Arevalo, 2020), as well as a different maturation rate with altered gene expression pattern during development (Rurak et al., 2022), and functional differences in physiological or inflammatory conditions (Santos-Galindo et al., 2011; Morizawa et al., 2012; Hasel et al., 2021). Interestingly, it seemed that overall, microglia and astrocytes have a more neuroprotective phenotypes in female rodents (Morizawa et al., 2012; Villa et al., 2018). To have a better understanding of the mechanisms and consequences related to neuroinflammation it appears necessary to consider sex as an important parameter.

Glial fibrillary acidic protein interleukin-6 (GFAP-IL6) mice over-express the cytokine IL6 only in astrocytes under the control of the *gfap* promoter, presenting an opportunity to assess the impact of chronic neuroinflammation on cognition, as well as associated behavioral domains and neuroinflammatory cascades (Campbell et al., 1993). IL6 has a complex role as it can be involved in both neuroprotection and neurodegeneration by activation of microglia and astrocytes (Okada et al., 2004; Garner et al., 2018; Kummer et al., 2021). GFAP-IL6 mice show age-dependent motor impairment (Campbell et al., 1993; Gyengesi et al., 2019; Asgarov et al., 2022) which corresponds with cerebellar volume loss, as well as elevated microglia and astrocytes in the hippocampus and cerebellum (Gyengesi et al., 2019; Ullah et al., 2020a). GFAP-IL6 mice show astrogliosis across the brain (Chiang et al., 1994; Ullah et al., 2020a,b), as well as synaptic disruptions in various brain regions including the frontal cortex, cerebellum, hippocampus (Heyser et al., 1997; Vereyken et al., 2007; Gyengesi et al., 2019).

However, the cognitive performance of these mice remains underexplored. GFAP-IL6 mice show impaired avoidance learning from 6 months of age (Heyser et al., 1997), and spatial memory recall impairment in the Barnes maze at 22, but not 8 months of age (Chesworth et al., 2021). Other behaviors which could impact on the expression of cognitive performance, e.g., exploration, anxiety, have not been assessed in GFAP-IL6 mice, although a recent study indicates 4 month old GFAP-IL6 homozygous mice exhibit greater depressive-like behavior in the forced swim test, decreased digging behavior and decreased exploratory behavior in the light/dark test compared to wildtype-like (WT) controls (Roberts et al., 2019). Considering the limited exploration of cognitive abilities in GFAP-IL6 mice, and the lack of exploration of other behavioral domains which could also impact on cognitive performance, e.g., anxiety, exploration; this study examined a range of cognitive domains, as well as exploratory and anxiety-like behaviors in more detail in GFAP-IL6 mice. As experiments to date using GFAP-IL6 mice have collapsed males and females (Heyser et al., 1997; Gyengesi et al., 2019; Ullah et al., 2020a,b; Chesworth et al., 2021), we sought to assess if females alone exhibited cognitive impairment. We used 11–15 month old animals as previous research has examined cognitive deficits in male and female GFAP-IL6 mice at 6 and 22 months (Heyser et al., 1997; Chesworth et al., 2021), but cognitive abilities within this large age range has not been examined. Finally, we also investigated gene expression of neuroinflammatory markers and downstream signaling pathways in brain areas highly relevant to motor function (i.e., cerebellum, motor cortex), as GFAP-IL6 mice have established motor deficits (Campbell et al., 1993; Gyengesi et al., 2019; Asgarov et al., 2022), as well as cognition (i.e., hippocampus, amygdala), which we explored in the present study.

## 2 Materials and methods

### 2.1 Animals

This study was performed on female heterozygous GFAP-IL6 mice (*n* = 12) and their female wild-type-like littermates (*n* = 15), aged between 11–15 months old. All animals were housed in individually ventilated cages (Type Mouse Version 1: Airlaw, Smithfield, Australia, with a floor area of 501 cm^2^) in groups of 2–4 animals per cage, with food and water *ad libitum* in the animal facility of the School of Medicine, Western Sydney University with a 12 h/12 h light/dark cycle (white lights on 8 am – 8 pm, red lights 8 pm – 8 am) under a controlled temperature of 22–25°C and humidity of 30–70%.

Environmental enrichment was provided and rotated every cage change, including gray polyvinyl chloride tubes, cardboard toilet paper rolls, red plastic domes, nesting material (tissues) and wooden gnawing sticks. All procedures were approved by the Animal Care and Ethics Committee (ACEC) of Western Sydney University (A12549) and were executed according to the “Guidelines to Promote the Wellbeing of Animals used for Scientific Purposes” established by the National Health and Medical Research Council of Australia.

### 2.2 Behavioral tests

**Figure 1:**
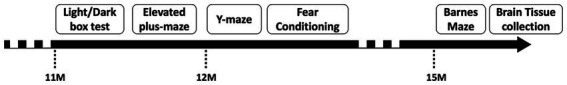
Behavioral test timeline including the tests and the age of the animals.

A behavioral test timeline is presented below (Figure 1). There was at least a 24–48 h inter-test interval between all tests. Animals were transferred from the animal facility to the testing room from 8 am (beginning of the light phase of the cycle). Mice were habituated to test rooms for 30 min prior to behavioral tests. For each test, the apparatus was cleaned with 80% ethanol v/v between each animal.

#### 2.2.1 Light/dark box test

The Light/Dark (LD) box apparatus was a modified open field activity chamber (43 × 43 cm, Med Associates, USA), and was equipped with an opaque dark box insert covering half the chamber. This separated a light zone (20 Lux) from a dark zone (< 2 Lux) by an opening in the center of the insert (9 cm wide).

Mice were placed in the dark compartment and allowed to explore freely for 10 min, during which horizontal and vertical activity was recorded using infrared photobeams (box size: 3; ambulatory trigger: 2; resting delay: 1,000 ms; resolution: 100 ms) (Kreilaus et al., 2021). Anxiety-like behavior was assessed by the time spent in and the distance traveled in the light vs. dark compartments (Crawley, 1985; Costall et al., 1989). Exploratory behavior (i.e., *rearing*) was measured via vertical activity (Kreilaus et al., 2021).

#### 2.2.2 Elevated plus-maze

The elevated plus-maze (EPM) was a PVC plus-shaped apparatus composed of two open arms (35 × 6 cm without side walls) and two closed arms (35 × 6 cm with 28 cm high side walls) connected by a central platform (6 × 6 cm). The surface of the arms was 70 cm from the floor. The lux level on the open arms was 30 lux. In this test, mice are naturally conflicted between exploration and avoidance of open elevated spaces (Lister, 1987). Greater time, entries and distance in the open arms of the maze indicates a lower level of anxiety-like behavior (Pellow et al., 1985).

In the EPM, mice were placed in the center of the maze, facing the closed arms, and were able to freely explore the apparatus for 5 min, according to published methods from our laboratory (Watt et al., 2020). The sessions were video recorded using AnyMaze™ tracking software, measuring entries, distance traveled, and time spent in the arms (Watt et al., 2020).

#### 2.2.3 Y-maze

The Y-Maze (YM) test was used to assess intermediate-term spatial memory (Contarino et al., 1999). The apparatus is an eight arm radial maze from which five arms remained closed to generate a Y-shaped maze (Stoelting Co., IL, USA). Each arm was 8 × 22 × 15 cm (L × W × H) and at 135°, 135°, and 90° from each other. Visual external cues (vertical stripes, horizontal stripes, plus-shape, circle and triangle) are displayed on the walls above the apparatus to provide spatial orientation (Kreilaus et al., 2021).

There were two trials of 10 min each with a 30 min intertrial interval, based on our laboratory’s published methods (Kreilaus et al., 2021). In the first trial, animals were placed at the center of the maze with only two arms open for exploration. In the second trial, animals were placed in the center of the apparatus and could now explore all three arms. The number of entries, time and distance traveled in each arm was recorded with AnyMaze™ tracking software, and data is presented as the time/distance/entries into the novel arm.

#### 2.2.4 Fear conditioning

Fear conditioning (FC) is a three day-test used to assess associative learning, which is dependent on the hippocampus and amygdala (Phillips and LeDoux, 1992; Radulovic and Tronson, 2010). In this test, animals learn to associate a neutral conditioned stimulus (i.e., tone, apparatus) with an aversive unconditioned stimulus (i.e., shock), which then elicits a fear response (i.e., *freezing*).

The apparatus was a mouse fear conditioning chamber (VFC-008, Med Associates, 29.5 cm × 24.5 cm × 21 cm), and methods have been published previously in our laboratory (Watt et al., 2020; Kreilaus et al., 2021). On day 1 (conditioning), mice were placed into the test chamber with vanilla scent for a 7 min trial. After 2 min, an 80 dB conditioned stimulus (5,000 kHz tone) was presented for 30 s with a 2 s co-terminating 0.4 mA foot shock; this occurred twice with an interval of 2 min. The test concluded 2 min later. On day 2 (context trial), mice were returned to the apparatus for 7 min with the vanilla scent cue present. On day 3 (cue trial), mice were placed into a distinct context for 9 min (no vanilla scent and a tent-like insert to create a novel environment). After 2 min, the tone was presented continuously for 5 min. The test concluded after another 2 min. *Freezing* behavior was measured by Video Freeze software (MED Associates Inc., VT, USA).

#### 2.2.5 Barnes maze

The Barnes Maze (BM) test is used to assess spatial learning and memory and is a dry land version of the Morris Water Maze (Barnes, 1979). The apparatus is composed of a circular platform 91 cm in diameter, elevated 90 cm above the floor, with 20 equally spaced holes of 5 cm diameter in the periphery. One of the holes is equipped with a small dark recessed chamber in which the mice can hide. Little plastic stairs were added to the chamber to facilitate access to the chamber. The other holes were obstructed. The position of the escape hole was randomly assigned for each mouse to minimize location bias. External visual cues were displayed around the apparatus, at 10 cm from the periphery of the platform (a star, a square, a circle, and horizontal stripes) as well as all around the room (a table, posters with black shapes, curtains). To motivate the mouse to escape, an aversive noise of 85 dB (2 kHz) and bright lighting (950 lux) were used during the test. All trials were 2 min and the intertrial interval was 1 h. BM procedures were conducted over 6 days. BM methods are based on our laboratory’s published work (Chesworth et al., 2021).

The first day (habituation) consisted of three trials. Test animals were placed at the center of the maze and allow to explore the apparatus freely without any aversive stimuli and with all holes obstructed.

The acquisition phase lasted for 4 days, with 3 trials per day. During acquisition, animals were first placed in a dark box at the center of the maze. The box was then lifted, the aversive noise was played, and the mouse was suddenly exposed to the bright light. The animal was allowed to explore the apparatus freely and the noise was turned off once the mouse entered the escape hole. If the mouse did not find and/or did not enter the escape hole before the end of the trial, it was gently guided into it. Mice were allowed to stay inside the escape hole for 30 s before being removed. The latency to find the escape hole was defined as ‘primary’ latency and the latency to enter the escape hole was defined as ‘secondary’ latency (Chesworth et al., 2021).

24-h after the final acquisition day, the mice were tested in a probe trial, which consisted of one trial with all holes obstructed (no escape hole). All sessions were recorded via AnyMaze™ tracking software and the latency to reach the target hole were measured.

We further evaluated how our animals learnt the location of the escape hole using a comprehensive search strategy analysis. For this, hole exploration was manually scored by an experimenter blind to genotypes. Based on previous literature examining search strategies in the BM (Harrison et al., 2006; Popović et al., 2010; O’Leary and Brown, 2012; Van Den Herrewegen et al., 2019), we defined 3 types of search strategy: Spatial: the mouse explored 2 or fewer holes before reaching/entering the target hole – Serial: the mouse visited at least 3 consecutive holes during the trial – Random: none of the previous definitions were applicable.

### 2.3 mRNA analysis

#### 2.3.1 Tissue collection

After completion of behavioral testing, mice (*n* = 10) were deeply anesthetized with isoflurane before being euthanized by cervical dislocation. Brains were harvested and placed in a brain matrix (ProSciTech, QLD, Australia), and single edge raor blades were used to generate 1 mm thick coronal sections. The razor blades were inserted in the matrix starting caudally of the olfactory bulb until the rostral part of the cerebellum. The hippocampus, amygdala, and motor cortex were sampled bilaterally using 0.96 mm puncher (Leica Biosystem, IL, USA). The whole cerebellum was collected (it wasn’t divided in 1 mm sections). All samples were collected in 1.7 mL Eppendorf tubes, flash frozen in liquid nitrogen and then stored at −80°C.

#### 2.3.2 RT-qPCR

Total RNA was extracted from the collected tissue using the Direct-zol™ RNA MiniPrep kit (Zymo Research, USA), following the manufacturer’s recommended protocol.

The RNA samples were first treated with DNase I recombinant, RNase free (Roche, Mannheim, Germany), then incubated at 37°C for 30 min, follow by 5 min at 75°C. The complementary DNA (cDNA) was synthetized using the ProtoScript® II first strand kit (New England BioLabs). The input quantity of RNA was 1 μg for the cerebellum (in a final volume of 20 μL). For the other regions (hippocampus, motor cortex and amygdala) the quantity of RNA extracted was too low to reach 1 μg, so the total volume eluted from the Direct-zol™ RNA MiniPrep kit column was used to generate cDNA (30 μL). First, a mix of 1.5 μg of oligo (dT) (Invitrogen, life technologies corporation, USA), 1.25 μg of Random hexamers (Invitrogen, life technologies corporation, USA) and a final concentration of 2.5 nM of each dNTP (dATP, dCTP, dGTP, dTTP) (Invitrogen, life technologies corporation, USA) was added to each RNA sample which were incubated at 65°C for 5 min. The samples were then put on ice and a mix of 5 μL of DTT 0.1 M, 6 μL of 5x Buffer, 4 μL of DNase/RNAse free water and 2 μL ProtoScript® II was added in each tube (except for the water, all products used were from the ProtoScript® II first strand kit, New England BioLabs). The samples were then incubated at 42°C for 50 min follow by 15 min incubation at 70°C. Samples were kept at −20°C.

SsoAdvanced SYBR® green from Supermix (BioRad, CA, USA) was used to perform RT-qPCR on a Mx3005P® apparatus (Stratagene, CA, USA). We used 0.8 μL of cDNA, 8 μL of primers (see list of primers in Table 1), and 20 μL of Supermix for a final volume of 40 μL that was divided in 3 wells (triplicates). The coding gene for Glyceraldehyde 3-phosphate dehydrogenase (GAPDH) was used as a housekeeping gene to normalize our results (its expression was stable across genotype) using the 2-ΔΔCt method (Livak and Schmittgen, 2001) and the fold change was centered on 0.

**Table 1:** Statistical analysis summary.

	Cerebellum			Motor cortex			Hippocampus			Amygdala		
	Mean	*t*	*p*	Mean	*t*	*p*	Mean	*t*	*p*	Mean	*t*	*p*
*Il6*	489.29	*t*_4_ = 3.7650	** 0.02	21.00	*t*_4_ = 3.4250	* 0.027	41.43	*t*_4_ = 18.5110	*** < 0.0001	13.20	*t*_4_ = 1.8610	ns 0.136
*Aif1*	6.66	*t*_4_ = 9.4380	*** 0.001	1.24	*t*_4_ = 7.6290	** 0.002	1.29	*t*_4_ = 6.9250	** 0.002	2.40	*t*_4_ = 3.9120	* 0.017
*Gfap*	24.51	*t*_4_ = 15.5570	*** < 0.0001	4.54	*t*_4_ = 6.7380	** 0.003	1.03	*t*_4_ = 6.4360	** 0.003	3.46	*t*_4_ = 4.0410	* 0.016
*Trem2*	4.97	*t*_4_ = 9.4410	*** 0.001	1.53	*t*_4_ = 6.7550	** 0.003	1.37	*t*_4_ = 4.8130	** 0.009	1.11	*t*_4_ = 2.8440	* 0.047
*Tspo*	4.64	*t*_4_ = 3.1920	* 0.033	1.44	*t*_4_ = 6.2420	** 0.003	1.57	*t*_4_ = 3.6540	* 0.022	0.61	*t*_4_ = 1.5130	ns 0.205
*P2x7*	0.97	*t*_4_ = 4.6010	** 0.01	0.45	*t*_4_ = 2.3130	ns 0.082	-			-		
*Il6ra*	5.24	*t*_4_ = 6.2600	** 0.003	0.65	*t*_4_ = 4.4470	* 0.011	0.57	*t*_4_ = 5.2210	** 0.006	−0.27	*t*_4_ = −0.9330	ns 0.403
*Gp130*	0.99	*t*_4_ = 6.0390	** 0.004	0.44	*t*_4_ = 2.5180	ns 0.066	-			-		
*Stat3*	2.25	*t*_4_ = 17.0710	*** < 0.0001	0.81	*t*_4_ = 5.4190	** 0.006	0.45	*t*_4_ = 3.5980	* 0.023	−0.02	*t*_4_ = −0.1170	ns 0.913
*NfkB*	1.20	*t*_4_ = 6.0290	** 0.004	0.40	*t*_4_ = 8.1320	*** 0.001	−0.32	*t*_4_ = −1.5350	ns 0.2	0.81	*t*_4_ = 2.5940	# 0.06
*Cox2*	10.84	*t*_4_ = 5.3600	** 0.006	0.24	*t*_4_ = 1.6080	ns 0.183	−0.53	*t*_4_ = −8.0220	*** 0.001	−0.17	*t*_4_ = −1.5220	ns 0.203
*Cas3*	0.45	*t*_4_ = 8.8370	*** 0.001	0.61	*t*_4_ = 2.6810	# 0.055	−0.22	*t*_4_ = −3.5050	* 0.025	−0.04	*t*_4_ = −0.1170	ns 0.913
*Nrf2*	1.90	*t*_4_ = 3.4360	* 0.026	–			–			–		
*Bdnf*	−1.75	*t*_4_ = −6.3630	** 0.003	−0.01	*t*_4_ = −0.0470	ns 0.965	–			–		
*Eno2*	−0.85	*t*_4_ = −10.0910	*** 0.001	0.32	*t*_4_ = 1.4750	ns 0.214	−0.19	*t*_4_ = −1.6070	ns 0.183	0.03	t_3_ = 0.2840	ns 0.795
*Gria2*	−0.71	*t*_4_ = −3.9370	* 0.017	–			–			–		
*Grin1*	−0.65	*t*_4_ = −3.5850	* 0.023	–			–			–		
*Slc6a1*	−0.24	*t*_4_ = −4.5820	** 0.01	–			–			–		
*Htr1a*	−0.80	*t*_4_ = −0.8780	ns 0.43	0.21	*t*_4_ = 1.3070	ns 0.261	0.19	*t*_4_ = 0.8120	ns 0.462	–		
*Ache*	0.01	*t*_4_ = 0.1830	ns 0.864	0.22	*t*_4_ = 1.3640	ns 0.244	0.02	*t*_4_ = 0.1290	ns 0.904	–		
*Slc1a2*	–			0.67	*t*_4_ = 3.9040	* 0.017	–			–		
*Chrna7*	–			0.22	*t*_4_ = 1.4760	ns 0.214	–			–		
*Gad1*	–			–			–			−0.03	*t*_4_ = −0.3070	ns 0.774
*Gabra5*	–			–			–			0.06	*t*_4_ = 0.6330	ns 0.561

The results are expressed as mean fold change centered in 0 (0 corresponds with no change), asterisks indicate differences compared to WT, *p ≤ 0.05, **p ≤ 0.01, ***p ≤ 0.001, #*p* ≤ 0.1.

### 2.4 Tissue preparation and immunohistochemistry

For histological analysis, the tissue samples were prepared from all experimental cohorts [methods (Gyengesi et al., 2019)]. Mice (*n* = 5) were anesthetized using isoflurane at the end of the behavioral testing and transcardially perfused. The heart was flushed with 60 mL of 0.1 M phosphate buffered saline (PBS) or 0.9% normal saline, followed by 100 mL of 4% paraformaldehyde (PFA) in 0.1 M PBS, using a peristaltic pump. The brains were then removed and post-fixed in 4% PFA, for at least 24 h at 4°C. Brains were then transferred to 30% sucrose (in 0.1 M PBS solution) for cryoprotection. Once the brains sank to the bottom of the container, they were cut into 50 μm thick coronal sections using a Leica CM 1950 cryostat. Six series of the sections were then stored in antifreeze solution (30% ethyleneglycol, 30% glycerol in phosphate buffered saline (PBS) and H_2_O) and stored at −20°C until use. The first series of the sections were used for immunohistochemical assays (Gyengesi et al., 2019) to visualize microglia and astrocytes. The sections were rinsed three times with 0.1 M PBS, and then incubated for 3 h in 3% normal donkey serum (Abcam, ab138579) (0.1 M PBS; 0.1% Triton X) to block the nonspecific binding sites. Sections were then incubated in primary antibody solution (rabbit anti-Iba1, 019–19,741, Wako Chemical, 1:500; mouse anti-GFAP-AF488, MAB3402X, Merck Millipore, 1: 500; 0.1 M PBS; 0.1% Triton X) for 48 h at 4°C. Sections were then rinsed with 0.1 M PBS for three times, and incubated in secondary antibody solution (Alexa Fluor 594-conjugated Affinity Pure donkey anti-rabbit IgG for Iba1, 1:200, 711-585-152 from Jackson Immuno Research) for 2 h at room temperature.

The second series of the sections were used to visualize activated neurons. The sections were rinsed three times with 0.1 M PBS, and then incubated for 3 h in 3% normal donkey serum (Abcam, ab138579) (0.1 M PBS; 0.1% Triton X) to block the nonspecific binding sites. Sections were then incubated in primary antibody solution (mouse anti-NeuN, #MA5-33103, ThermoFisher, 1:500; rabbit anti-cFos, #PA-830, ThermoFisher, 1: 500; 0.1 M PBS; 0.1% Triton X) for 48 h at 4°C. Sections were then rinsed with 0.1 M PBS for three times, and incubated in secondary antibody solution (Alexa Fluor 594-conjugated Affinity Pure donkey anti-mouse IgG and Alexa Fluor 488-conjugated Affinity Pure donkey anti-rabbit IgG, 1:200, Jackson Immuno Research) for 2 h at room temperature.

Sections were then rinsed and mounted on gelatine-coated slides, and cover slipped with Vectashield mounting medium hard set with DAPI (Vectorlabs, H-1500). Representative images were taken using a Zeiss AxioImager M2 microscope.

### 2.5 Stereological counting

The estimated number of Iba-1^+^ microglial cells and GFAP^+^ astrocytes in the cerebellum and the hippocampus were counted using the Zeiss AxioImager M2 microscope equipped with MBF Biosciences StereoInvestigator (West et al., 1991). The contour of the cerebellum and hippocampus was first drawn under the 5x objective. The size of the counting frame was 100 × 100 μm for wild type animals (for both areas analyzed), and 60 × 60 μm for GFAP-IL6 animals (for both areas analyzed). The counting grid was 1,500 × 1,000 μm for the cerebellum and 800 × 800 μm for the hippocampus for all cohorts. The guard zone was 1 μm at the top and the bottom of the sections. Microglia and astrocytes were plotted on the screen using different markers as the focus moved from the top to the bottom of the sections using a 63× oil objective. This led to a Gunderson coefficient error of less than 0.1 in all cases (*m* = 1).

### 2.6 Statistics

One or two-way ANOVA, as well as RM ANOVA with Bonferroni *post hoc* tests (when relevant) and single sample *t*-tests were performed to analyze the behavioral results using IBM® SPSS® software. Huynh-Feldt correction was used when necessary. The degree of fold change for mRNA was analyzed with *t*-tests for each marker separately, using IBM® SPSS® software. The estimated number of microglia and astrocytes in both the cerebellum and the hippocampus were compared between groups using unpaired *t*-test in Graphpad Prism 10. Results were considered significant if *p* < 0.05; *p*’s between 0.05–0.1 were considered a trend.

## 3 Results

### 3.1 Chronic overexpression of IL6 in the CNS leads to a less anxious-like phenotype in mice

**Figure 2:**
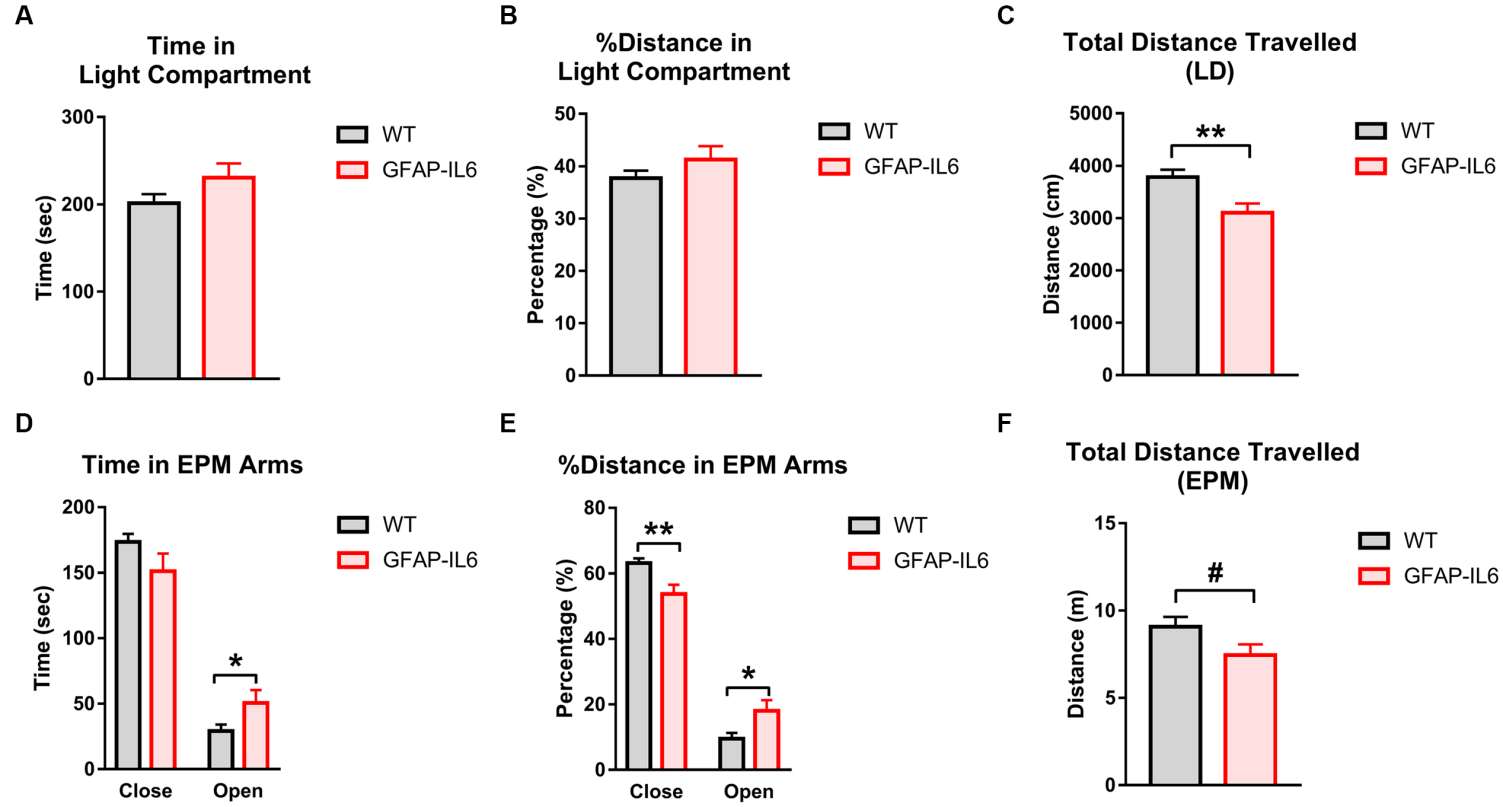
GFAP-IL6 mice exhibit an anxiolytic, hypolocomotor phenotype compared to WT mice. **(A)** Time (s) spent in the Light compartment, **(B)** percentage (%) of distance traveled in the Light compartment, **(C)** total distance travelled (cm) in the LD, **(D)** time (s) spent in the EPM arms, **(E)** distance travelled (m) in the EPM, **(F)** total distance travelled (m) in the EPM in WT and GFAP-IL6 mice. Data analyzed with one-way ANOVA, and presented as means ± S.E.M. Main effects of ‘genotype’ indicated by asterisks (*p < 0.05, **p < 0.01), trends by hash symbols (#*p* = 0.06) (*n* = 15 WT;12 GFAP-IL6).

The LD and EPM were used to assess anxiety-like behavior. In the LD, there were no differences between GFAP-IL6 and WT mice for the time in the light zone (Figure 2A) or percentage of distance travelled in the light zone (Figure 2B). Latency to enter the light zone and light zone entries were also unaffected by GFAP-IL6 genotype (data not shown). In the EPM, GFAP-IL6 mice spent more time in the open arms than WTs [main effect of genotype; *F_(1,26)_* = 4.363, *p* = 0.047] (Figure 2D). In the EPM, as a percentage of total distance, GFAP-IL6 mice traveled less in the closed arms [main effect of genotype; *F_(1,26)_* = 9.847, *p* = 0.004] and more in the open arms [main effect of genotype; *F_(1,26)_* = 5.841, *p* = 0.023] compared to WT animals (Figure 2E).

Total distance travelled was reduced in GFAP-IL6 mice compared to WT controls in the LD [main effect of genotype; *F_(1,26)_* = 9.502, *p* = 0.005] (Figure 2C), and there was a trend for this in the EPM as well [main effect of genotype; *F_(1,26)_* = 3.843, *p* = 0.061] (Figure 2F). This suggests that GFAP-IL6 mice display a less anxiety-like behavior than WT mice and reduced locomotion.

### 3.2 Chronic IL6 overexpression impairs intermediate-term spatial memory but not associative learning and memory

**Figure 3:**
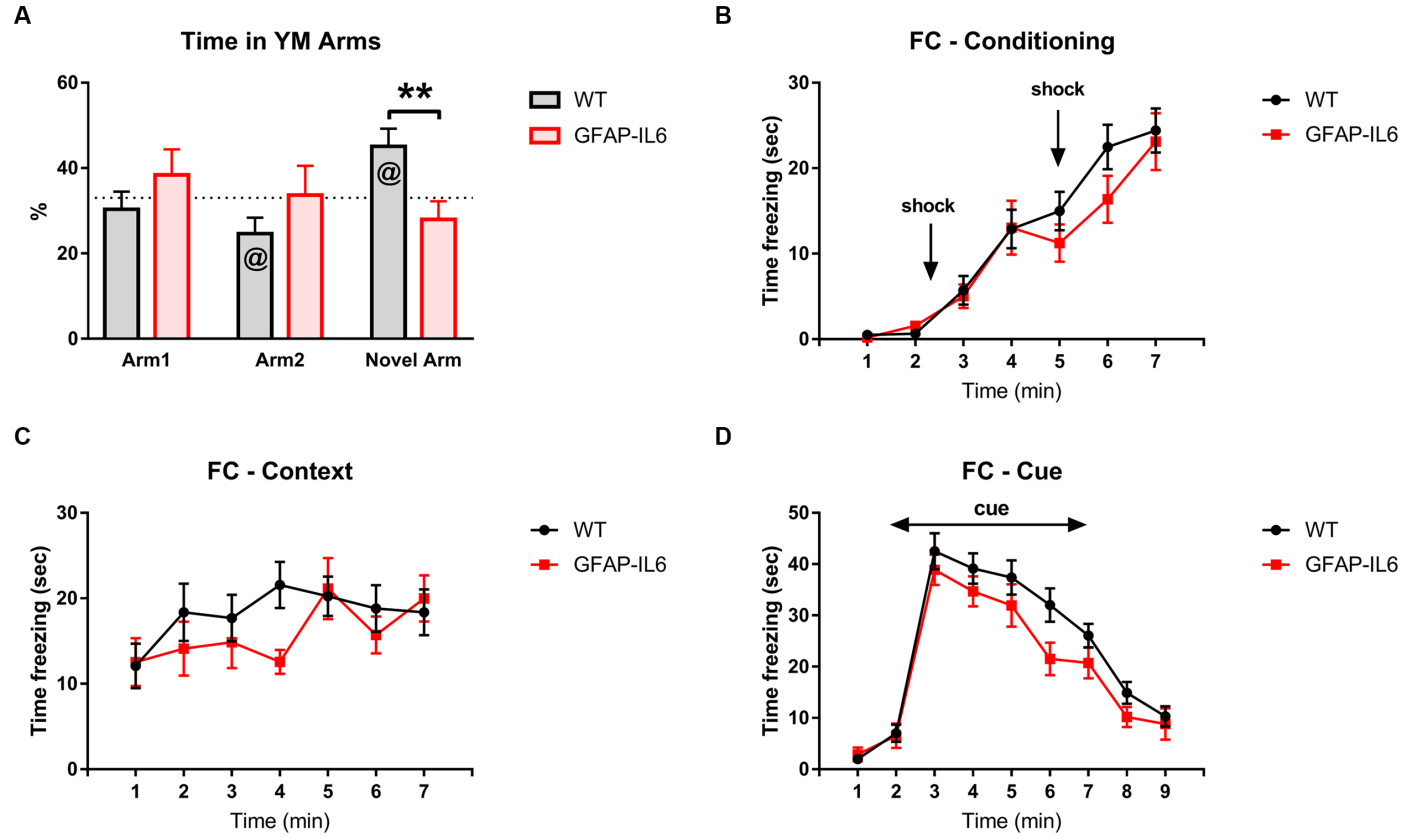
GFAP-IL6 mice display impaired intermediate-term memory but not associative learning and memory. **(A)** Percentage (%) of time spent in the arms of the YM by GFAP-IL6 and WT mice. Time (s) ‘*freezing’* for FC during **(B)** Conditioning Day, **(C)** Context Day, and **(D)** Cue Day for GFAP-IL6 and WT mice. Data analyzed with one-way ANOVA and one-sample *t*-test (theoretical value = 33) for the YM and two-way RM ANOVA for the FC, and presented as means ± S.E.M. Main effect of ‘genotype’ indicated by asterisks (**p < 0.01), and significant difference from theoretical value in the *t*-test indicated by ‘at’ symbols (^@^*p* ≤ 0.05) (*n* = 15 WT;12 GFAP-IL6).

To assess intermediate-term spatial memory learning and memory, animals were tested in the Y-Maze (YM). As a proportion of total time, GFAP-IL6 mice spent less time in the novel arm than WT mice [*F_(1,26)_* = 8.093, *p* = 0.009] (Figure 3A). WT mice spent more time in the novel arm compared to chance, but GFAP-IL6 mice did not [WT Novel arm: *t_14_* = 2.905, *p* = 0.12; GFAP-IL6 Novel arm: *t_11_* = −1.181, *p* = 0.263] (Figure 3A). These results suggest an impaired intermediate-term spatial memory in the YM in GFAP-IL6 mice compared to WT mice.

We also assessed amygdalo-hippocampal-based learning and memory in GFAP-IL6 mice using fear conditioning. We analyzed the time spent *freezing* during conditioning, context, and cue days. For all tests, *freezing* changed across the test sessions, indicating memory of the tone-shock pairings [two way RM ANOVA, main effect of time, Conditioning: *F_(6,150)_* = 53.307, *p* < 0.001; Context: *F_(6,150)_* = 3.087, *p* = 0.007; Cue: *F_(8,200)_* = 77.405, *p* < 0.001] (Figures 3B–D), but this was not different between WT and GFAP-IL6 mice [no main effect of genotype – Conditioning: *F_(6,150)_* = 0.696, *p* = 0.412; Context: *F_(6,150)_* = 0.676, *p* = 0.419; Cue: *F_(8,200)_* = 2.659, *p* = 0.116; no interactions between time and genotype]. Overall, these results suggest that GFAP-IL6 mice have impaired intermediate-term spatial memory but not associative learning and memory.

### 3.3 Chronic IL6 overexpression contributes to longer secondary latencies and different search strategy profiles in the BM

**Figure 4:**
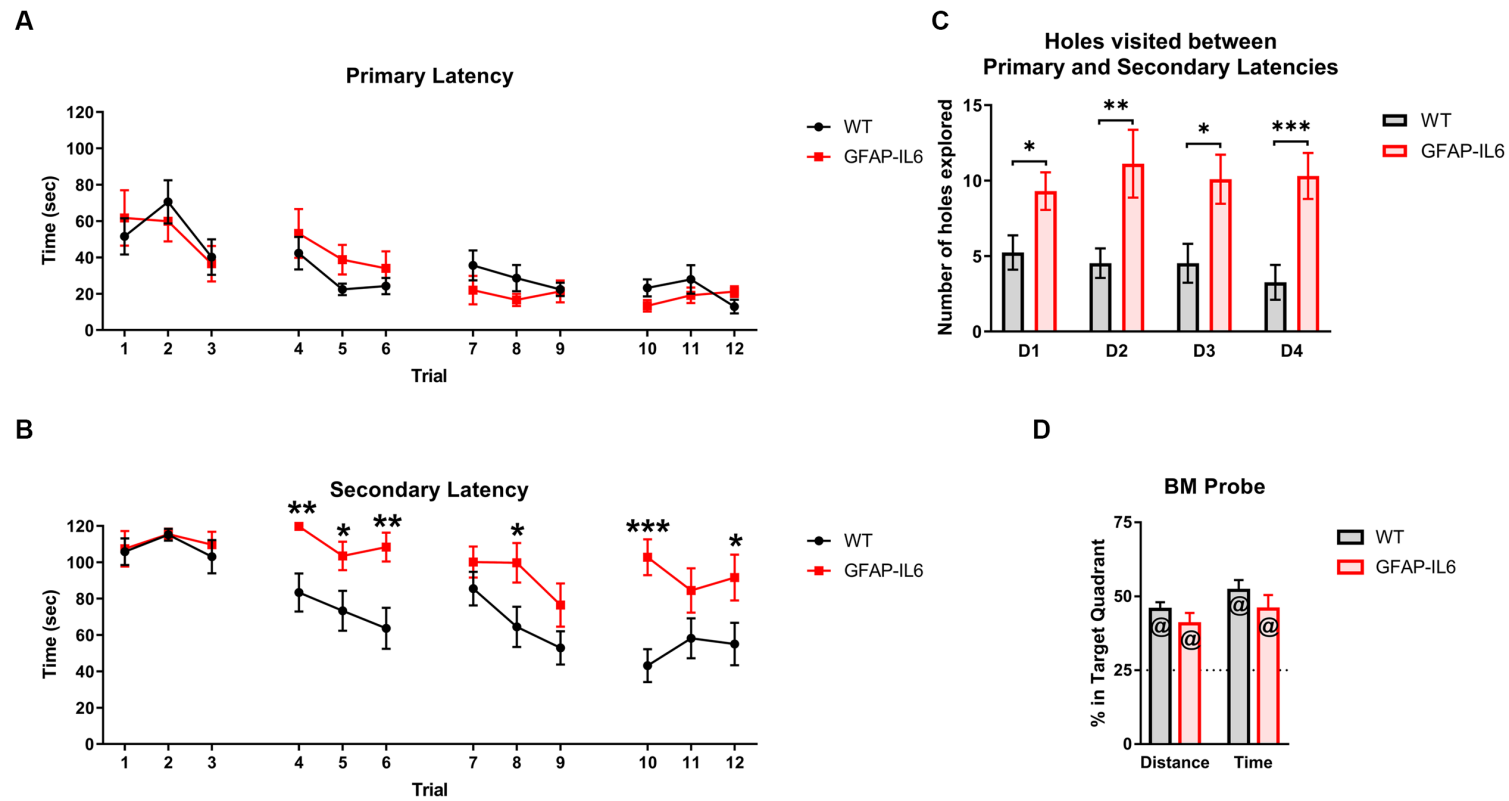
GFAP-IL6 mice learn the location of the escape hole but exhibit higher secondary latencies in the Barnes Maze. **(A)** Primary latencies (latency to find the escape hole), (s) and **(B)** Secondary latencies (latency to enter the escape hole), (s) for GFAP-IL6 and WT mice during BM acquisition days. **(C)** Number of holes (*n*) explored by GFAP-IL6 and WT mice after the primary latency and before the secondary latency. **(D)** Percentage (%) of Distance travelled and Time spent in the target quadrant by GFAP-IL6 and WT mice during the BM probe test. Data presented as means ± S.E.M and analyzed with two-way RM ANOVA for the latencies, and one-way ANOVA and one-sample *t*-test (theoretical value = 25) for probe test measures. Significant differences between genotypes after post-hoc analysis (Bonferroni) indicated by asterisks (*p < 0.05, **p < 0.01, ***p < 0.001), and significant difference from theoretical value in the *t*-test indicated by ‘at’ symbols (^@^*p* ≤ 0.05) (*n* = 14 WT; 11 GFAP-IL6).

To further explore spatial learning and memory in our animals we used the BM. The primary latency significantly decreased during acquisition trials [main effect of trials: *F_(11,253)_* = 7.874, *p* < 0.001] with no genotype differences [no main effect of genotype: *F_(11,253)_* = 0.006, *p* = 0.938; no interactions] (Figure 4A), suggesting that all mice learnt the location of the escape hole. The secondary latency also decreased during acquisition trials [main effect of trials: *F_(11,253)_* = 7.864, *p* < 0.001], however, GFAP-IL6 mice exhibited higher secondary latencies to enter the escape hole compared to WT mice [main effect of genotype: *F_(1,23)_* = 10.842, *p* = 0.003; genotype x trial interaction: *F_(11,253)_* = 2.389, *p* = 0.008] (Figure 4B). After finding the escape hole for the first time but before entering it, the GFAP-IL6 also showed a higher level of exploration as they visited more holes than the WT mice on each test day (averaged across trials, Figure 4C) [main effect of genotype: *F_(1,23)_* = 17.289, *p* < 0.001].

During the probe test, WT and GFAP-IL6 mice both travelled further and spent more time in the target quadrant (i.e., more than 25% of the total time and total distance travelled) (Figure 4D) [Distance: WT *t_13_* = 7.938, *p* < 0.001, GFAP-IL6 *t_10_* = 3.842, *p* = 0.003; Time: WT *t_13_* = 7.762, *p* < 0.001, GFAP-IL6 *t_10_* = 4.477, *p* = 0.001] but again there was no genotype differences for these measures [no main effect of genotype: *F_(1,24)_* = 0.404, *p* = 0.531].

**Figure 5:**
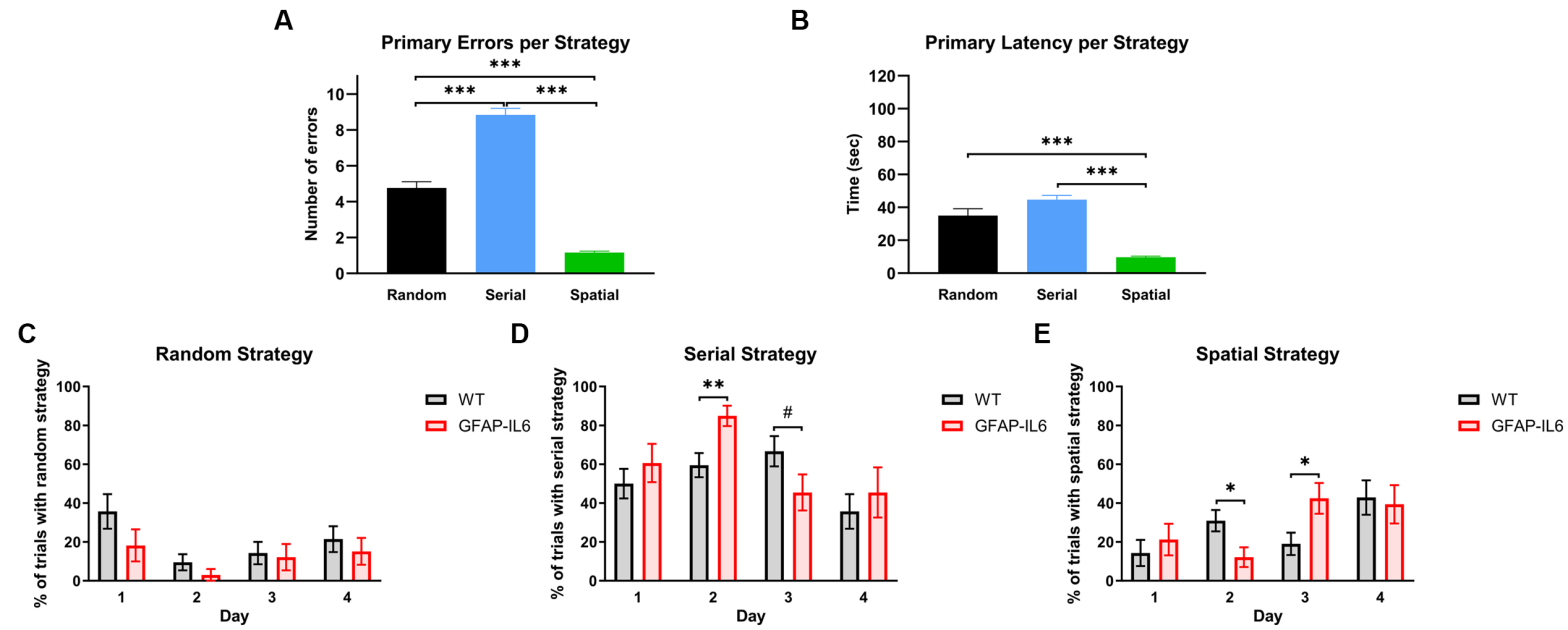
GFAP-IL6 and WT mice use different search strategies across days, and these strategies have different efficiencies. **(A)** Primary Errors (N) and **(B)** primary Latencies (s) per search strategy. Percentage (%) of trials per day in which WT and GFAP-IL6 mice used **(C)** ‘Random’, **(D)** ‘Serial, or **(E)** ‘Spatial’ search strategies. Data presented as means ± S.E.M and analyzed with one-way ANOVA for **A,B** and two-way RM ANOVA for **C–E**. Significant differences between search strategies and genotype after post-hoc analysis (Bonferroni) indicated by asterisks (*p < 0.05, ***p < 0.001) (*n* = 14 WT;11 GFAP-IL6).

We decided to further analyze the BM acquisition results by looking at the search strategy used by the animals to locate the escape hole. To verify our definition we analyzed the number of primary errors (Figure 5A) and primary latencies (Figure 5B) based on the search strategy used, to evaluate the efficiency of the search strategies. We found a significant effect of the search strategy used for primary latency [*F_(2,299)_* = 43.458, *p* < 0.001] and primary errors [*F_(2,299)_* = 122.494, *p* < 0.001]. As per the definition, the number of primary errors for the ‘Spatial’ strategy was significantly lower compared to the other search strategies [post-hoc comparison Bonferroni – Spatial vs. Random: *p* < 0.001, Spatial vs. Serial: *p* < 0.001] (Figure 5A). The ‘Spatial’ strategy led to the lowest primary latency [Post-hoc comparison Bonferroni – Spatial vs. Random: *p* < 0.001, Spatial vs. Serial: *p* < 0.001] (Figure 5B). There was no significant differences in the primary latencies between the ‘Random’ and ‘Serial’ Strategy [post-hoc comparison Bonferroni – Random vs. Serial: *p* = 0.097] (Figure 5B). However, there were significantly more primary errors in the ‘Serial’ strategy compared to the ‘Random’ strategy [post-hoc comparison Bonferroni – Random vs. Serial: *p* < 0.001] (Figure 5A). This means that the animals using the ‘Serial’ strategy were able to find the escape hole as fast as the animals using the ‘Random’ strategy.

We then looked at the search strategy used each day before reaching the primary latency. All animals used the ‘Random’ strategy (Figure 5C) more on the first day of acquisition compared to the second day [two-way RM ANOVA main effect of day: *F_(3,69)_* = 3.332, *p* = 0.024]. This was not different between the genotypes [no main effect of genotype: *F_(1,23)_* = 3.189, *p* = 0.087]. Animals used the ‘Serial’ strategy (Figure 5D) differently across days [main effect of day: *F_(3,69 – Huynh-Feldt corr.)_* = 5.080, *p* = 0.004] which also depended on the genotype [day x genotype interaction: *F_(3,69 – Huynh-Feldt corr.)_* = 2.918, *p* = 0.043]. On day 2, GFAP-IL6 mice used the ‘Serial’ strategy more than the WT mice [Post-hoc comparison Bonferroni – WT Day 2 vs. GFAP-IL6 Day 2: *p* = 0.006] and this pattern tended to be inverted on day 3 [Post-hoc comparison Bonferroni – WT Day 3 vs. GFAP-IL6 Day 3: *p* = 0.092]. The progression of the use of ‘Spatial’ strategy (Figure 5E) seem to be complementary to what happened for the ‘Serial’ strategy. There was a significant effect of day [*F_(3,69)_* = 4.133, *p* = 0.009] as well as a significant day x genotype interaction [*F_(3,69)_* = 2.992, *p* = 0.037] suggesting that each genotype used the spatial strategy differently across days. Pairwise comparisons showed that WT animals used the spatial strategy significantly more than GFAP-IL6 on day 2 [Post-hoc comparison Bonferroni – WT Day 2 vs. GFAP-IL6 Day 2: *p* = 0.022], while GFAP-IL6 mice used the spatial strategy more than WTs on day 3 [Post-hoc comparison Bonferroni – WT Day 3 vs. GFAP-IL6 Day 3: *p* = 0.022]. Inspection of Figures 5C–E shows that GFAP-IL6 and WT animals both mainly used the random search strategy on the first day and increased their use of the spatial strategy as they reached the 4^th^ day of acquisition. However, the genotypes showed different learning strategies (i.e., serial vs. spatial) from days 2–4. It seems that the GFAP-IL6 mice ‘switched’ from serial strategy on day 2 to the spatial strategy on day 3, which seemed to happen only in the WT mice as they reached day 4, even though the WT mice compared to the GFAP-IL6 mice were already using the most efficient strategy (spatial strategy) on day 2.

### 3.4 mRNA analysis shows increased pro-inflammatory markers in various brain regions of GFAP-IL6 mice

**Figure 6:**
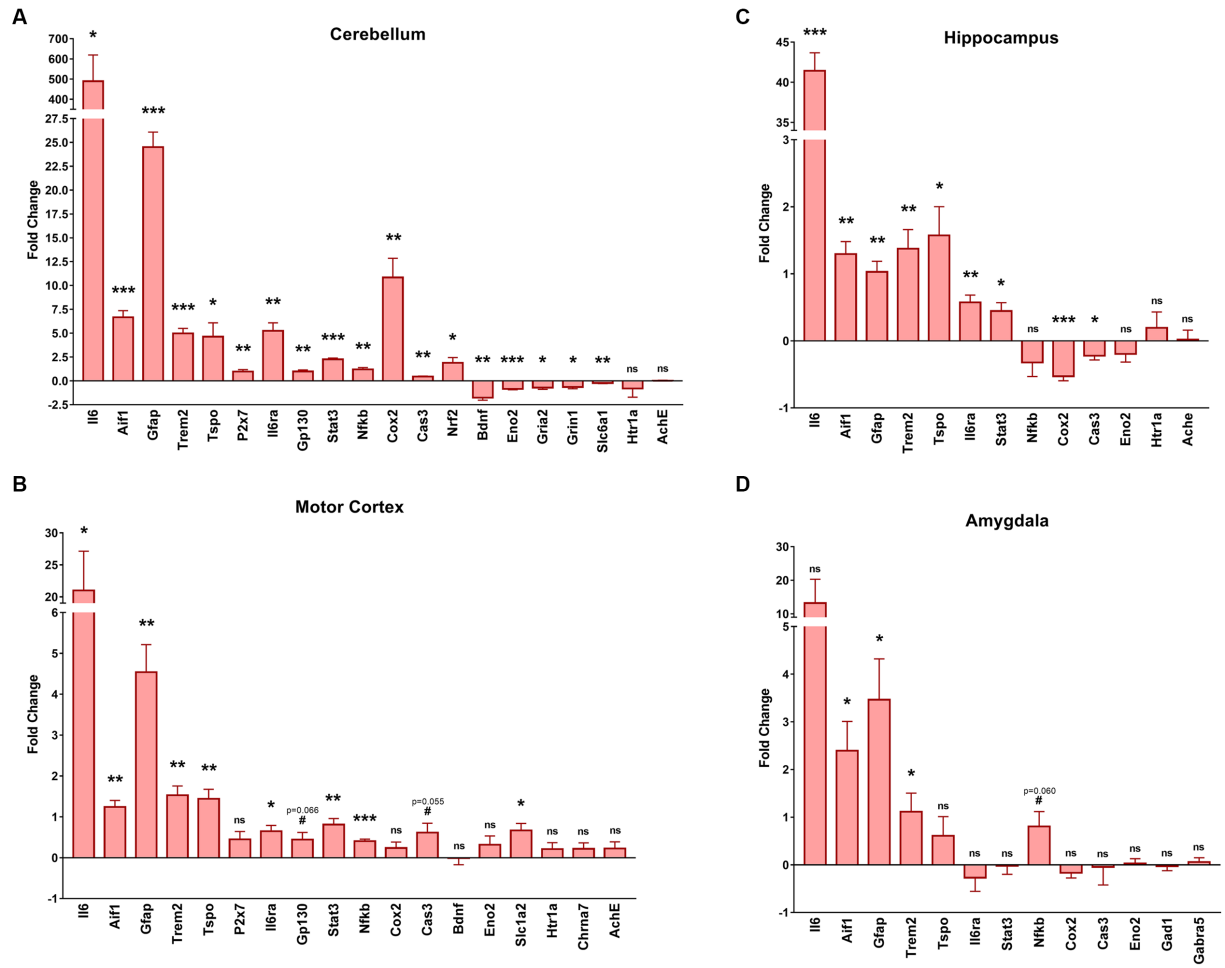
GFAP-IL6 mice exhibit various transcriptomic changes in the cerebellum, motor cortex, hippocampus and amygdala. We measured via RT-qPCR the mRNA levels of various genes involved in microglial activation, pro-inflammatory pathways, or neurotransmission in four different brain regions including the cerebellum **(A)**, the motor cortex **(B)**, the hippocampus **(C)** and the amygdala **(D)**. The results are expressed as fold change compared to WT, centered at 0 (0 corresponds with no change). *Gapdh* was used as a housekeeping gene. Significant genotype differences are indicates by asterisks, *p ≤ 0.05, **p ≤ 0.01, ***p ≤ 0.001, #*p* ≤ 0. 1. vs. WT.

We extracted the mRNA from several brain regions: the cerebellum (Figure 6A), motor cortex (Figure 6B), hippocampus (Figure 6C) and amygdala (Figure 6D), and compared the expression level of genes related to neuroinflammation, neurodegeneration, and neurotransmission between GFAP-IL6 and WT mice. The results are presented as fold change (FC) in GFAP-IL6 (compared to WTs) and the statistical analysis is summarized in Table 1.

In all regions, we found increased mRNA levels for *Aif1* (ionized calcium-binding adapter molecule 1, Iba1), and *Trem2* (triggering receptor expressed on myeloid cells 2) which are related to microglial activation, as well as increased mRNA levels of *Gfap* which is associated with astrogliosis. The *Eno2* (Enolase-2, neuronal marker) gene was only downregulated in the cerebellum and unchanged for the other 3 regions. For the cerebellum, hippocampus, and motor cortex there was an increased transcription of *Tspo*, another marker of microglial activation, *Ilra* (IL6 Receptor), *Stat3* (signal transducer and activator of transcription 3) and *IL6* which are all involved in IL6 signaling. For the cerebellum, there was also an increase of *P2x7* (P2X purinoceptor 7) mRNA, a purinergic receptor involved in microglial activation that wasn’t observed in the motor cortex, as well as an upregulation of *Gp130* (which facilities signal transduction following IL6 expression) that was only found as a tendency for the motor cortex (*t*_4_ *p* = 0.066). Interestingly the pro-inflammatory downstream nuclear factor *Nfκb* (nuclear factor kappa-light-chain-enhancer of activated B cells) was only slightly but significantly upregulated in the cerebellum (FC = 1.2) and the motor cortex (FC = 0.4) but not in the hippocampus, and with only a tendency (*t*_4_ *p* = 0.06) to be upregulated in the amygdala. The amygdala was the region for which we found the fewest changes in gene transcription, with no significant transcriptional change of *Tspo* (translocator protein), *Il6ra*, *Stat3, Cox2* (cyclooxygenase-2), *Cas3* (caspase 3), *Eno2*, *Gad1* (glutamate decarboxylase 1), *Gabra5* (gamma-aminobutyric acid A receptor α5) or even *IL6*. Interestingly, *Cox2* and *Cas3* were both upregulated in the cerebellum, but downregulated in the hippocampus and were not changed in the motor cortex even though we observed a tendency for *Cas3* upregulation (*t*_4_ *p* = 0.055). There was no change in *Htr1a* (5-hydroxytryptamine Receptor 1A) or *Ache* (acetylcholine esterase) expression in the cerebellum, motor cortex, or hippocampus. In the motor cortex, there was no evidence of changes to expression of *Bdnf* (brain derived neurotrophic factor) or *Chrna7* (α7 nicotinic acetylcholine receptor) but there was an upregulation of the *Slc1a2* (glutamate transporter 1) gene. In the cerebellum we found an upregulation of *Nrf2*, an anti-inflammatory marker, and a downregulation of several genes involved in GABAergic and glutamatergic neurotransmission such as *Slc6a1* (GABA transporter 1), *Grin1* (N-methyl-D-aspartate 1 receptor – NMDA1) and *Gria2* (α-amino-3-hydroxy-5-methyl-4-isoxazolepropionic acid receptor 2 – AMPA2) as well as a downregulation of *Bdnf*. We found no significant differences in the expression of our housekeeping gene (*Gapdh*) between genotypes.

### 3.5 Increased glial expression in the GFAP-IL6 animals can be observed in the cerebellum and the hippocampus

**Figure 7:**
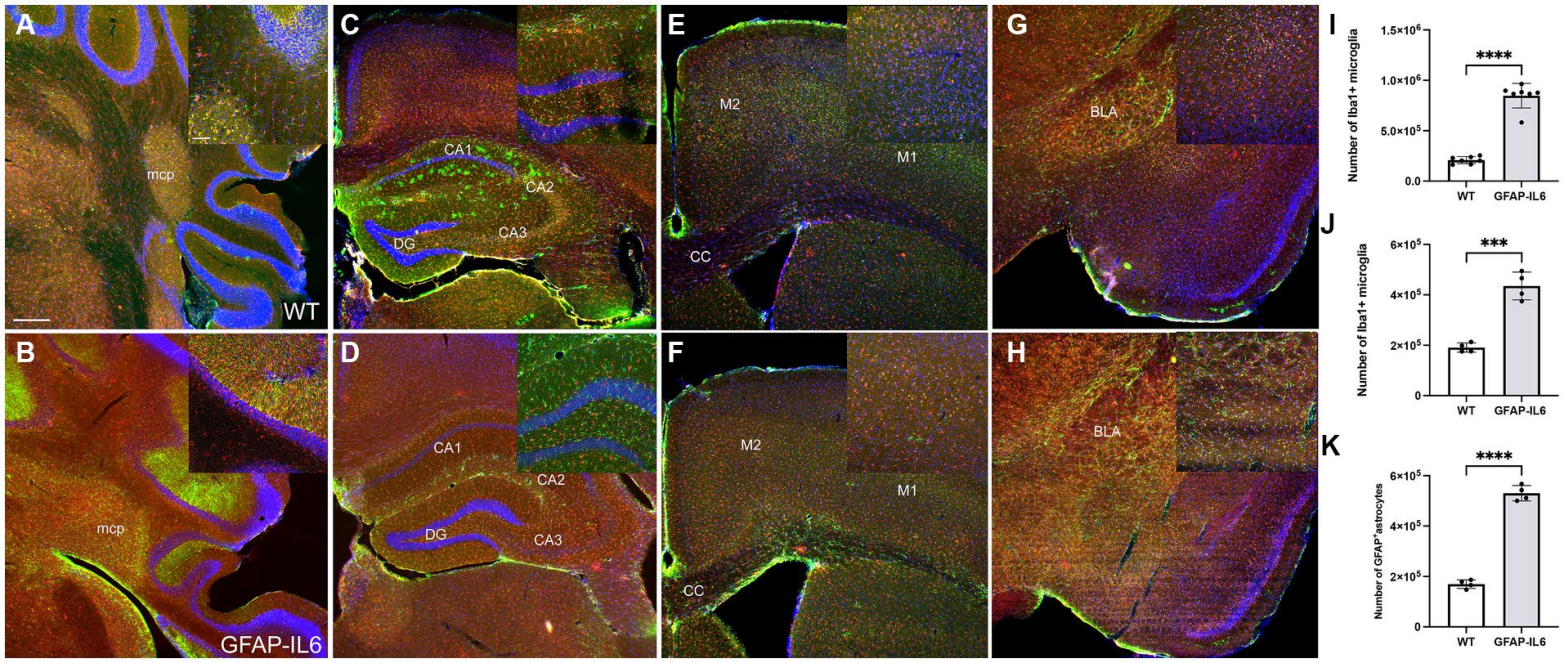
GFAP-IL6 mice exhibit increased gliosis in the cerebellum and the hippocampus, evidenced by astroglial and microglial activation. Representative images of WT **(A,C,E,G)** and GFAP-IL6 **(B,D,F,H)** showing the expression of Iba-1 (microglia marker) and GFAP (astroglia marker) in the cerebellum **(A,B)**, the hippocampus **(C,D)**, the motor cortex **(E,F)** and the amygdala **(G,H)**. Increased glial expression is evident in the GFAP-IL6 females in the cerebellum and hippocampus, and we can see a slight increase of astroglial expression in the amygdala. Note, the GFAP antibody used for this staining fails to label astrocytes in the cortex. Cerebellar microglial **(I)** and hippocampal microglial **(J)** and astroglial **(K)** numbers were estimated using stereological counting, showing consistent and significant increase in the GFAP-IL6 females compared to their wild type counterparts (***p < 0.001, ****p < 0.0001). Scale bar represents 500 μm on the main panel and 50 μm on the inserts. Significant main effects of ‘genotype’ are indicated by asterisks (****p < 0.0001).

To test the effect of chronic glial activation on microglial and astroglial populations in female mice, immunohistochemistry and stereological counting of Iba-1^+^microglia and GFAP^+^ astrocytes was performed in the hippocampus and cerebellum in wild type and GFAP-IL6 mice after the completion of the behavioral tests. We observed increased gliosis indicated by microglial and astroglial markers in the GFAP-IL6 animals in areas of the cerebellum, hippocampus (Figures 7A–D), but less noticeable differences in the motor cortex and the amygdala (Figures 7E–H). In the cortex, the expression pattern of the microglia seems similar between the genotypes. Note that the expression of astrocytes using this antibody cannot be compared in the cortical areas, as this particular antibody does not label cortical astrocytes.

In the cerebellum, we found a significant difference in microglial numbers between genotypes [*F*(6,6) = 12.1, *p* < 0.0001]. We found that GFAP-IL6 mice had 847,457 ± 122,023 Iba-1^+^ microglia, which was significantly higher than that of the wild type WT mice (208,128 ± 35,008) (*p* < 0.0001) (Figure 7I). Due to the morphological changes of the cerebellar GFAP^+^ astrocytes, we were unable to quantify their numbers, but it is evident that a high level of gliosis is taking place, resulting in rod shaped astroglial morphology. Similarly, in the hippocampus, we found a significant difference in both microglial and astroglial numbers between genotypes [*F*(3,3) = 9.12, *p* < 0.0001], [*F*(3,3) = 3.19, *p* < 0.0001], respectively. We found that GFAP-IL6 mice had 435,069 ± 55,037 Iba-1^+^ microglia, which was significantly higher than that of WT the wild type mice (190,090 ± 18,222) (*p* < 0.0001) (Figure 7J).

We found that GFAP-IL6 mice had 530,375 ± 30,448 GFAP^+^ astrocytes, which was significantly higher than that of the wild type WT mice (169,908 ± 17,047) (*p* < 0.0001) (Figure 7K).

**Figure 8:**
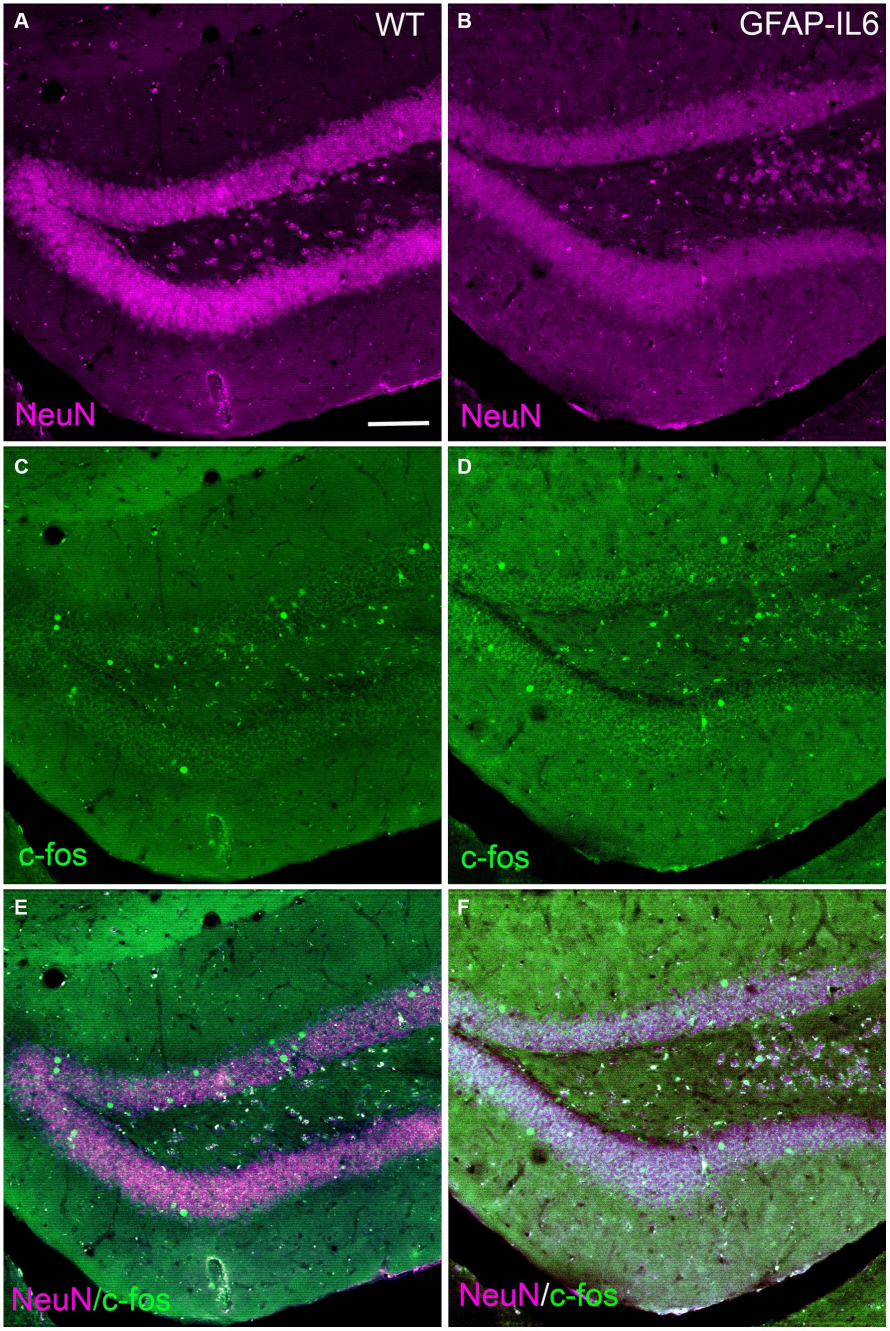
Representative images of neurons and c-Fos activation in the dentate gyrus of WT and GFAP-IL6 mice. Representative images of WT **(A,C,E)** and GFAP-IL6 **(B,D,F)** showing the expression of NeuN (neuronal marker, magenta) and c-Fos (cell activation, green) in the dentate gyrus of the hippocampus. Scale bar represent 50 μm.

In addition, to investigate if neuron numbers in the hippocampus, specifically in the dentate gyrus or activity using c-Fos in the dentate gyrus, a structure involved in encoding new experiences, was altered in the GFAP-IL6 mice, we performed immunohistochemistry for NeuN and c-Fos markers. We found c-Fos expressing cells in both WT and GFAP-IL6 mice, in both hemispheres of the brain. The neuronal marker NeuN expression did not seem to differ between genotypes. Qualitative analysis of the sections did not show evident changes in these cell markers at this age (Figure 8).

## 4 Discussion

In our study, the constitutive overexpression of IL6 in the CNS in aged female heterozygous GFAP-IL6 mice led to the dysregulation of anxiety-like behavior, locomotor activity and spatial learning and memory. Compared to WT mice, GFAP-IL6 mice showed reduced anxiety-like behavior in the EPM, hypolocomotion in the LD test, and short-term spatial memory impairment in the YM. However, GFAP-IL6 mice showed intact associative learning and memory in FC. Spatial learning and memory in the BM also appeared unaffected in GFAP-IL6 when analyzing primary latencies, but secondary latencies were higher in GFAP-IL6 mice, potentially reflecting decreased anxiety. Our search strategy analysis uncovered differences in the way GFAP-IL6 mice searched for and thus learnt the escape hole location. To better understand the behavioral phenotype observed in GFAP-IL6 mice and it’s association with molecular changes triggered by chronic neuroinflammation, we explored transcriptomic changes in various brain regions: the cerebellum, motor cortex, hippocampus and amygdala. Chronic exposure of the brain to IL6 had different effects on each brain region. Of the brain regions examined by RT-qPCR, the cerebellum showed the greatest number of changes to neuroinflammatory, neurodegenerative, and neurotransmission markers, while the amygdala showed the least number of changes in GFAP-IL6 compared to WT mice. However, all regions showed a significant upregulation of mRNA levels for molecules involved in pro-inflammatory pathways in the GFAP-IL6 mice, validating our model our chronic neuroinflammation.

Motor deficits from 6 months of age are consistently observed in GFAP-IL6 heterozygous mice (Campbell et al., 1993; Gyengesi et al., 2019; Asgarov et al., 2022). We found a similar phenotype in 11 month old female GFAP-IL6 mice, with reduced distance travelled in the LD and a trend for this in the EPM, although more detailed motor function tests, e.g., beam walking, accelerod, ataxia would confirm this phenotype more thoroughly. Hypolocomotion in GFAP-IL6 mice aligns with the fact that the cerebellum shows the highest level of IL6 expression in this model (Campbell et al., 1993; Chiang et al., 1994; Gruol et al., 2014), and that GFAP-IL6 mice show significant age-dependent cerebellar volume loss (Gyengesi et al., 2019), impaired blood brain barrier permeability at the cerebellar level (Brett et al., 1995; Campbell et al., 2014), and the highest level of microglial activation and astrogliosis in the cerebellum compared to other brain regions (Gyengesi et al., 2018; Ullah et al., 2020b). Furthering this, here we found that the cerebellum showed the greatest degree of gene expression fold change, to the greatest number of markers. All pro-inflammatory markers either reflecting microglial or astroglial activation and possible proliferation, or genes involved in the IL6 pro-inflammatory cascade were upregulated, which was also evident in the histological results, with Iba-1 and GFAP expression strongly upregulated in the cerebellum and the hippocampal areas. The reported microglial and astroglial numbers in this female cohort was not significantly different from our previously published estimated cell numbers of the cerebellum and hippocampus in mixed sexes cohorts. Interestingly there was also an upregulation of the anti-inflammatory marker *Nrf2*, suggesting that there is still an anti-inflammatory response in the cerebellum which may not be strong enough to protect the cerebellum from the consequences of chronic neuroinflammation as, for example, the neuroprotective factor *Bdnf* was downregulated. We also found an upregulation of *Cas3*, a marker of apoptosis, and the downregulation of *Eno2* (a neuronal marker), which could be related to the cerebellar volume loss observed in GFAP-IL6 mice (Gyengesi et al., 2019). Considering *Gria2*, *Grin1*, and *Slc6a1* are largely expressed in neurons, it is possible that the downregulation of *Gria2*, *Grin1*, and *Slc6a1* could be due to a loss of neurons (related to the downregulation of *Eno2*) rather than a downregulation of their transcription in the neurons. We also noted that almost all the pro-inflammatory markers were upregulated as well in the motor cortex. Compared to the results in the cerebellum, we only observed a tendency for *Cas3* upregulation and no significant change for *Eno2*. In the motor cortex, there was a downregulation of *Slc1a2*, which codes for the glutamate transporter 1, suggesting an impairment of glutamatergic neurotransmission in the motor cortex and more specifically a deficit of glutamate clearance by the astrocytes and a potential excito toxicity that could be further investigated.

Short term spatial learning and memory in female GFAP-IL6 heterozygous mice was impaired in the YM. Despite this, amygdalohippocampal dependent learning and memory assessed by FC was intact. This was surprising as cellular and molecular changes in GFAP-IL6 heterozygous mice have been observed extensively in the hippocampus and, to a lesser extent, in the amygdala (Samland et al., 2003; Gruol et al., 2014; Roberts et al., 2019; Ullah et al., 2020b; West et al., 2022), which would suggest that their function might be impaired. Nonetheless, it is possible that test parameters may limit the expression of cellular and molecular changes in the amygdala and hippocampus on cognition. For example, FC produces a robust memory which is recalled for up to 2 months post-training (Pickens et al., 2009), but YM spatial memory lasts for 24 h (Hughes, 2004) and it is possible that a less robust memory is more susceptible to learning and memory impairment induced by chronic neuroinflammation in GFAP-IL6 mice. Supporting this, deficits in YM spatial memory are linked to elevated hippocampal inflammatory markers (e.g., Iba1, NFκB) (Kong et al., 2020), and we also found clear upregulation of neuroinflammatory and glial activation markers such as *Aif1*, *Gfap*, *Trem2*, *Tspo*, *Stat3* and *Nfkb* in the hippocampus, suggesting chronic neuroinflammation in mice can impair some types of learning and memory in the YM. Interestingly, there was a small (FC = 0.53) but very significant (*p* = 0.001) downregulation of *Cox2*. As *Cox2* is not only related to neuroinflammation but also to neuronal firing and memory (Minghetti, 2004; López and Ballaz, 2020), analysis of more genes involved in this cascade is necessary to understand this change, as its downregulation could be related to YM spatial memory impairment. Furthermore, confirmation of the YM deficit with other short-term spatial memory tasks and also analysis of other ages in female GFAP-IL6 heterozygous mice would support this finding.

Our results revealed that 11 month old female GFAP-IL6 heterozygous mice present a test-dependent anxiolytic phenotype, whereby GFAP-IL6 mice showed increased time in the open arms of the EPM, but no changes to time spent in the light zone of the light–dark test. The test parameters in these experiments might explain the different phenotypes between the two tests of anxiety-relevant behavior. Indeed, the EPM is more anxiety-inducing than the light–dark test due to the combination of the elevated platform, an open space and a higher lux level on the open arms compared to the light compartment of the light–dark test. Interestingly, the decrease in anxiety-like behavior in the EPM was also observed in 1 month old mice where IL6 was overexpressed via an adenovirus injected intraventricularly at birth (Wei et al., 2012). This supports our data and suggests that early and chronic overexpression of IL6 can decrease anxiety-like behaviors. Despite differences in anxiety-like behavior in GFAP-IL6 female mice, we found few changes to gene expression in the amygdala. There was evidence of microglial and astroglial activation and possible proliferation as *Aif1*, *Gfap* and *Trem2* were upregulated. However, the transcription of other genes such as *Gad1*, involved in GABA production or *Gabra5*, GABAergic receptor, or even *Cas3* or *Eno2* was not significantly changed, and IL6 was also not significantly upregulated. Downstream effects of IL6 overexpression may account for the decreased anxiety-like phenotype observed. The glutamate transporter, which was decreased in the motor cortex, may play a role in the decreased anxiety-like behavior observed here, as increasing glutamate transporter activity in astrocytes via ceftriaxone administration increases open arm time in mice (Alajaji et al., 2013; Hammad et al., 2021) and reduces the expression of neuroinflammatory factors, e.g., NFĸB, TNF-α (Hammad et al., 2021). Together with our data, this suggests a potential bidirectional relationship between neuroinflammation and glutamate transporter 1 function. Future evaluation of glutamate transporter mRNA expression in the amygdala and medial prefrontal cortex of GFAP-IL6 mice could help to explain the decreased anxiety-like behavior in female GFAP-IL6 mice.

There was no long-term spatial learning and memory impairment in the BM in 15 month old GFAP-IL6 females. Interestingly, previous findings from our laboratory (Chesworth et al., 2021) reported spatial memory recall impairment at 22 months of age in GFAP-IL6 mice (mixed sexes). It is possible that 15 months may be too early to observe age-aggravated cognitive decline, as we have also shown that there is no spatial learning and memory impairment in GFAP-IL6 mice of mixed sexes in the BM at 8 months (Chesworth et al., 2021). In the current study, the secondary latencies, or latencies to enter the escape hole were significantly higher in GFAP-IL6 mice. In the BM, the mice are motivated to find the escape hole by anxiety: a bright and loud open arena is the incentive for the mouse finding the escape hole. Thus, greater secondary latencies support the finding of reduced anxiety-like behavior in these animals, which was evident in the EPM. Despite motor impairments in GFAP-IL6 mice [see LD data and also (Campbell et al., 1993; Gyengesi et al., 2019; Asgarov et al., 2022)], this did not prevent GFAP-IL6 mice from exploring more holes than WT mice after reaching the correct escape hole. Our analysis of search strategy suggests there are subtle changes in the use of spatial strategies between the genotypes, where WT mice use spatial strategies more often than GFAP-IL6 mice earlier during training. This suggests a subtle shift in spatial learning which may be an early indicator of the spatial learning and memory deficits observed at 22 months of age in GFAP-IL6 mice of both sexes (Chesworth et al., 2021). Future analysis of search strategies at later ages in GFAP-IL6 mice, and also comparing both sexes may reveal more pronounced changes in search strategy approach with aging.

In addition, it has been shown that IL-6 is regulated by estrogen, suggesting that elevated IL-6 levels may contribute to neuroinflammation and dementia particularly in women (Kurebayashi et al., 1997; Gao et al., 1998; Cerciat et al., 2010; Zhu et al., 2021). In the mouse brain, IL-6 deficiency was associated with lower cerebellar myelin basic protein (*p* < 0.05) levels (Miller et al., 2010). In addition also, in old-aged IL-6 deficient males had higher GFAP and MDA levels (*p* < 0.05) in both the hippocampus and cerebellum, in addition to a greater body weight than WT (Miller et al., 2010). These results suggested that IL-6 is important for promoting myelin synthesis in aged females, and that drugs which inhibit the synthesis of IL-6 in males may inadvertently affect fatty acid metabolism and augment aging-related neuroinflammation (Miller et al., 2010). Interleukin-6 was shown to contribute to sex differences in microglial function in the brain as well (Garrido-Gil et al., 2022). The impact of glial activation on neuronal numbers needs to be further investigated in this mouse model to provide a carefully analyzed quantitative description.

Taken these together, glial function changes might be one of the underlying mechanisms related to sex differences which warrants further investigations on how IL6 contributes via to behavioral changes in this mouse model during aging.

## 5 Conclusion

Chronic overexpression of IL6 in the CNS decreases motor function, produces a less anxious-like phenotype and impairs short term spatial learning and memory in the YM in the 11–15 month old female GFAP-IL6 heterozygous mice. Gene expression analysis demonstrates that GFAP-IL6 female mice express an increased inflammatory load, resulting in signs of impaired neuronal homeostasis. Taken together, female GFAP-IL6 mice appear to be a suitable model of chronic neuroinflammation with which to test novel anti-inflammatory compounds.

## Data availability statement

The original contributions presented in the study are included in the article/supplementary material, further inquiries can be directed to the corresponding author.

## Ethics statement

The animal study was approved by Western Sydney University Animal Care and Ethics Committee (A14644). The study was conducted in accordance with the local legislation and institutional requirements.

## Author contributions

IW: Conceptualization, Data curation, Formal analysis, Methodology, Visualization, Writing – original draft, Writing – review & editing. LJJ: Data curation, Methodology, Visualization. GN: Supervision, Writing – review & editing. GM: Conceptualization, Funding acquisition, Project administration, Writing – review & editing. TK: Resources, Supervision, Validation, Writing – review & editing. RC: Conceptualization, Formal analysis, Methodology, Resources, Supervision, Validation, Writing – review & editing. EG: Conceptualization, Funding acquisition, Project administration, Resources, Supervision, Writing – review & editing.
